# Characterization of the Mutagenic Spectrum of 4-Nitroquinoline 1-Oxide (4-NQO) in *Aspergillus nidulans* by Whole Genome Sequencing

**DOI:** 10.1534/g3.114.014712

**Published:** 2014-10-27

**Authors:** Damien J. Downes, Mark Chonofsky, Kaeling Tan, Brandon T. Pfannenstiel, Samara L. Reck-Peterson, Richard B. Todd

**Affiliations:** * Department of Plant Pathology, Kansas State University, Manhattan, Kansas 66506; † Department of Cell Biology, Harvard Medical School, Boston, Massachusetts 02115; ‡ Faculty of Health Sciences, University of Macau, Taipa, Macau SAR, China

**Keywords:** filamentous fungi, genetic screen, chemical mutagenesis, 4-nitroquinoline 1-oxide, whole genome sequencing

## Abstract

4-Nitroquinoline 1-oxide (4-NQO) is a highly carcinogenic chemical that induces mutations in bacteria, fungi, and animals through the formation of bulky purine adducts. 4-NQO has been used as a mutagen for genetic screens and in both the study of DNA damage and DNA repair. In the model eukaryote *Aspergillus nidulans*, 4-NQO−based genetic screens have been used to study diverse processes, including gene regulation, mitosis, metabolism, organelle transport, and septation. Early work during the 1970s using bacterial and yeast mutation tester strains concluded that 4-NQO was a guanine-specific mutagen. However, these strains were limited in their ability to determine full mutagenic potential, as they could not identify mutations at multiple sites, unlinked suppressor mutations, or G:C to C:G transversions. We have now used a whole genome resequencing approach with mutant strains generated from two independent genetic screens to determine the full mutagenic spectrum of 4-NQO in *A. nidulans*. Analysis of 3994 mutations from 38 mutant strains reveals that 4-NQO induces substitutions in both guanine and adenine residues, although with a 19-fold preference for guanine. We found no association between mutation load and mutagen dose and observed no sequence bias in the residues flanking the mutated purine base. The mutations were distributed randomly throughout most of the genome. Our data provide new evidence that 4-NQO can potentially target all base pairs. Furthermore, we predict that current practices for 4-NQO−induced mutagenesis are sufficient to reach gene saturation for genetic screens with feasible identification of causative mutations via whole genome resequencing.

4-Nitroquinoline 1-oxide (4-NQO) is a highly carcinogenic chemical that causes mutations in bacteria, fungi, and animals. 4-NQO has been used widely in the study of DNA damage and DNA repair and to generate mutants for genetic screens. 4-NQO induces mutagenesis after metabolic conversion to 4-hydroxyaminoquinolone 1-oxide (4-HAQO) (Miller 1970), which forms stable bulky adducts on purines (Tada and Tada 1976). Based on *in vitro* studies as well as in *Escherichia coli* and animal cells, 4-HAQO forms the majority of adducts (~50%) on the second nitrogen (N2) of guanine (Tada and Tada 1971; Galiegue-Zouitina *et al.* 1986; Bailleul *et al.* 1989). However, carbon eight (C8) guanine adducts (Bailleul *et al.* 1981; Galiègue-Zouitina *et al.* 1984; Tada *et al.* 1984) and nitrogen six (N6) adenine adducts (Galiegue-Zouitina *et al.* 1985, 1986) also occur at a lower frequency, ~30% and ~10%, respectively (Bailleul *et al.* 1989). Additional lesions were thought to be caused by production of reactive oxygen species (Kohda *et al.* 1986). In *E. coli* and mammalian cells, 4-HAQO adducts are repaired by the nucleotide excision repair pathway (Ikenaga *et al.* 1975a,b, 1977; Ikenaga and Kakunaga 1977), and in *E. coli* the error prone DNA polymerase IV (Pol IV) is the likely cause of sequence changes (Williams *et al.* 2010). Early work to characterize the mutagenic effects of 4-NQO in the yeasts *Saccharomyces cerevisiae* and *Schizosaccharomyces pombe*, as well as in the bacteria *Salmonella typhimurium* and *E. coli*, relied upon reversion of characterized auxotrophic tester strains, as DNA sequencing technology was not yet readily available (Prakash *et al.* 1974; Janner *et al.* 1979; Rosenkranz and Poirier 1979). These experiments identified the changes induced by 4-NQO as G:C to A:T transitions, G:C to T:A transversions, and frameshifts (Prakash *et al.* 1974; Janner *et al.* 1979; Rosenkranz and Poirier 1979). However, differences in frequency and mutation type varied between species and with 4-NQO concentration (Rosenkranz and Poirier 1979). Studies relying on reversion tester strains are limited by their inability to detect or determine multiple mutations in the same target gene as well as unlinked suppressor mutations, and the lack of strains to specifically detect G:C to C:G transversions (Prakash and Sherman 1973). In addition, these strains were not informative as to how flanking sequence affects mutagenic potential. Furthermore, auxotrophic reversion tester strains may show mutational bias due to functional constraints. Therefore, the full 4-NQO mutagenic spectrum, including type and relative frequency of induced mutations as well as the effect of flanking sequence, remains to be determined.

The genetic model filamentous fungus *Aspergillus nidulans* has been invaluable for advances in understanding a variety of eukaryotic cellular processes, including cell-cycle progression, development, response to DNA damage and pH changes, gene regulation, and metabolism (Clutterbuck 1969; Arst and Cove 1973; Morris 1975; Oakley and Oakley 1989; Harris *et al.* 1994; Goldman and Kafer 2004; Penalva *et al.* 2008; Wong *et al.* 2008). Many of these advances have been made using genetic screens. The versatility of *A. nidulans* for genetic analysis is due to several amenable characteristics, including stable haploid and diploid life stages as well as asexual and sexual reproduction (Pontecorvo *et al.* 1953). Heterozygous diploid strains, constructed via the parasexual cycle, can be used for analysis of dominance or complementation and to map novel mutations to a chromosome by haploidization (Todd *et al.* 2007a). Mutations can then be mapped more finely by classical genetic mapping via the sexual cycle (Todd *et al.* 2007b). Furthermore, the well-developed DNA-mediated transformation system, with homologous gene targeting and multiple selectable markers, enables construction of strains for mutational analysis and selection of mutants in genetic screens, and reconstruction of identified candidate mutations to identify the causative mutation associated with the mutant phenotype (Nayak *et al.* 2006). *A. nidulans* has been used extensively in genetic screens for mutants generated by a variety of chemical and physical mutagens, including *N*-methyl-*N*′-nitro-*N*-nitrosoguanidine (MNNG) (Clutterbuck 1969; Hynes and Pateman 1970a,b; Arst and Cove 1973; Osman *et al.* 1993), nitrous acid (Apirion 1965; Clutterbuck 1969), diethyl sulfate (Clutterbuck 1969), ultraviolet (UV) light (Pontecorvo *et al.* 1953; Clutterbuck 1969; Axelrod *et al.* 1973; Morris 1975; Osman *et al.* 1993), and X-rays (Pontecorvo *et al.* 1953). However, many genetic screens in *A. nidulans* use 4-NQO (Harris *et al.* 1994; Wu *et al.* 1998; Pokorska *et al.* 2000; Conlon *et al.* 2001; Heck *et al.* 2002; Kinghorn *et al.* 2005; Cecchetto *et al.* 2012; Larson *et al.* 2014; Tan *et al.* 2014) because it is safer and more stable than MNNG and it is thought to produce primarily single base-pair substitutions, which can generate both loss-of-function and altered function mutants. These altered function mutants are important for identifying essential genes in which larger mutations would be lethal. The utility and application of 4-NQO as a mutagen in genetic screens highlight the importance of understanding the full consequences of 4-NQO mutagenesis.

To fully characterize the mutagenic potential of any chemical, analysis of mutations that are unbiased by the selection method or gene function is required. A genomics approach, rather than sampling a single gene target by reversion of auxotrophies, overcomes limitations imposed by functional constraints, as mutations in noncoding regions and mutations unrelated to the selection and independent of function also can be detected. Whole genome sequencing has been used to identify the effects of ethyl methanesulfonate, ethylnitrosourea, and UV light in several eukaryotes, including *Arabidopsis thaliana* (Uchida *et al.* 2011), *Danio rerio* (Voz *et al.* 2012), *Caenorhabditis elegans* (Flibotte *et al.* 2010), and the apicomplexan parasite *Toxoplasma gondii* (Farrell *et al.* 2014). Recent advances in sequencing technology have permitted rapid and affordable resequencing of fungal genomes, and this has enabled identification of causative mutations in mutants generated in genetic screens (McCluskey *et al.* 2011; Pomraning *et al.* 2011; Nowrousian *et al.* 2012; Bielska *et al.* 2014; Tan *et al.* 2014; Yao *et al.* 2014; Zhang *et al.* 2014). In this work, we have used a genome resequencing approach to fully characterize the 4-NQO mutagenic spectrum at a whole genome level using almost 4000 4-NQO−induced mutations arising from independent genetic screens (Tan *et al.* 2014; this study). 4-NQO causes all possible base-pair substitutions with a 19-fold preference for guanine over adenine residues.

## Materials and Methods

### *A. nidulans* strains, media, growth conditions

*A. nidulans* strains RT244 (*biA1 pyrG89 gpdA*(p)*areA*^HA^ *fmdS-lacZ pyroA4 nkuA*∆*::Bar* [*prnA::areA*^NES^*::gfp::AfpyroA*] *crmA*^T525C^*::pyrG*) and RPA520 (*yA::*[*gpdA*(p)mCherry::FLAG::PTS1::*Afpyro*] *pabaA1 pyrG89* [TagGFP2::*rabA::AfpyrG*] *pyroA4 nkuA*∆*::argB* [HH1::TagBFP::*Afribo*]) were used for mutagenesis. Mutant strains generated from RPA520 were outcrossed to RPA478 (*pyrG89* [TagGFP2::*rabA::AfpyrG*] *pyroA4 nkuA*∆*::argB* [HH1::TagBFP*::Afribo*] *riboB2*) or RPA496 (*pyrG89* [TagGFP2*::rabA::AfpyrG*] *pyroA4 nkuA*∆*::argB* [HH1::TagBFP*::Afribo*]). *A. nidulans* growth conditions and media adjusted to pH 6.5 were as described (Cove 1966). *Aspergillus* nitrogen-free minimal media containing 1% w/v glucose and nitrogen sources (ammonium tartrate, sodium nitrate, or L-proline) added to a final concentration of 10 mM (Cove 1966), or rich yeast and glucose media (Szewczyk *et al.* 2006), supplemented for auxotrophies, were used for growth.

### Mutagenesis and sequencing

Mutagenesis using 4-NQO (Sigma-Aldrich) was carried out primarily as described (Holt and May 1996; Tan *et al.* 2014). In summary, ~10^7^ or ~10^8^ conidia, suspended in phosphate buffer (0.1 M potassium phosphate pH 7.0, 0.01% Tween 80) and quantified using a hemocytometer, were exposed to 0.24−4.0 µg mL^−1^ 4-NQO at 37° for 30 min. 4-NQO was quenched with an equal volume of 0.5 M sodium thiosulfate and washed twice in phosphate buffer. Strains were recovered from 50%, 10%, and 3% survival treatments (0.24 µg mL^−1^ 4-NQO per 10^7^ spores, 0.45 µg mL^−1^ 4-NQO per 10^7^ spores, and 4.0 µg mL^−1^ 4-NQO per 10^8^ spores, respectively) after 2−4 days’ growth on either yeast and glucose media or supplemented *Aspergillus* nitrogen-free minimal media containing 10 mM L-proline and tested for mutant phenotypes. Proline-using mutant phenotypes in strains derived from RT244 were mapped by meiotic crossing to RT250 (*yA1 pabaA1 pyrG89 gpdA*(p)*areA*^HA^ *fmdS-lacZ prn-309*). Genomic DNA was isolated as described (Lee and Taylor 1990). The genomes of RT244 and a derivative mutant strain were sequenced by the Genome Sequencing Facility (Kansas University Medical Center, Kansas City, Kansas) on an Illumina HiSEQ 2500 platform using single-end 50-bp reads. The genomes of RPA478, RPA496, RPA520, and bulked segregrant progeny of derivative mutant strains were sequenced by single-end, whole genome sequencing on the Illumina Genome Analyzer HiSeq 2000 platform, generating sequence reads ~50 base pairs in length (Tan *et al.* 2014). For mutant strains from RT244 showing tight linkage of the causative mutation and *prnA*, the mutations were identified by amplification of the *prnA*::*areA*^NES^::*gfp* regions with prn3′-F (5′-TCACGGCTATTCCGTGCTTTGA-3′) and gfp5′-R (5′-ACGCTGAACTTGTGGCCGTTA-3′) using Ex Taq (TaKaRa) and sequencing at Kansas State University DNA Sequencing and Genotyping Facility.

### *In silico* analysis

*In silico* analysis used the Galaxy platform (galaxyproject.org) (Blankenberg *et al.* 2010b) and Broad Genome Analysis Toolkit (GATK; broadinstitute.org/gatk) (McKenna *et al.* 2010). FASTA files were converted to FASTQ format using FASTQ Groomer (Blankenberg *et al.* 2010a). Sequence quality was determined using FastQC (Li and Durbin 2009) (bioinformatics.babraham.ac.uk/projects/fastqc/). Nucleotide sequence reads were aligned using Burrows-Wheeler Alignment for Illumina with default settings to the *A. nidulans* FGSC_A4 genome (Version S10) downloaded from AspGD (Cerqueira *et al.* 2014). Genome coverage was determined using BEDTools (Quinlan and Hall 2010). Sequence coverage was lacking or not aligned for the centromeres, the ribosomal rRNA repeats, and mitochondrial sequences. Variants were identified using FreeBayes (Garrison and Marth 2012) with default settings except for report polymorphism probability (−P: 0.01), ploidy (−p: 1), minimum observations (−F: 0.5), and minimum coverage (−!: 4) or using GATK (Depristo *et al.* 2011) with default settings except for quality score >50 (−stand_call_conf: 50.0, −stand_emit_conf: 10.0) and down sampling to 50 fold coverage (−dcov: 50.0). Variants unique to mutant strains were identified using Select Variants (Depristo *et al.* 2011). Aligned sequence reads from wild-type strains were manually inspected to confirm the absence of all identified unique variants. Box plots were generated using JMP 11 (SAS), outliers in boxplots are points lying 1.5 × interquartile range (third quartile to first quartile) above the third quartile or below the first quartile. The Student’s *t*-test and simple χ^2^ test were computed in Excel (Microsoft Office). SAS 9.4 (SAS) was used for exponential quantile-quantile plots (CAPABILITY procedure: QQplot / exponential, σ = est, θ = est), Kolmogorov-Smirnov tests (UNIVARIATE procedure with histogram & exponential settings), and categorical χ^2^ tests (FREQ and GENMOD procedures). Consensus motifs of mutated sites were generated using WebLogo (Crooks *et al.* 2004) (weblogo.berkeley.edu). *A. nidulans* sequence annotation of transcribed and intergenic regions, and gene function descriptions were obtained from AspGD (Cerqueira *et al.* 2014), and descriptions of yeast orthologs were obtained from SGD (Cherry *et al.* 2012).

### Prediction of saturation

We derived the following random sampling with replacement equation that can be adjusted to calculate the probability of a specific mutation of every nucleotide (nucleotide saturation) or every possible substitution at every nucleotide (substitution saturation):

$$P_{S\left(X\right)}={\left(1-{\left(1-f.b^{-1}\right)}^{m.s.\left(1-k\right)}\right)}^{b}$$

The standard equation for probability of a specific event (**X**) given multiple random samples with replacement is P_(X)_ = 1 − (1 − N^-1^)^n^, where **N^−1^** is the probability of the specific event given a single sample was taken, and **n** is the number of samples taken. For our equation, N^−1^ is replaced with the relative frequency with which a specific mutation arises (**f**) divided by the total number of base pairs at which it could have arisen (**b**). The number of samples is the mean number of mutations arising per spore (**m**), multiplied by the number of treated spores (**s**), multiplied by the number of surviving spores (**1 − k**), where k is the proportion kill, *i.e.*, for a mutation at a single base-pair P_S(X)_ = 1-(1-f.b^-1^)^m.s.(1-k)^. To determine the probability of a mutation at every possible base pair, where the likelihood of mutating any base pair is equivalent due to random mutagenesis, the probability of a single event is raised to the power of the number of base pairs (**b**), giving the final equation **P_S(X)_** for the probability of saturation of a specific mutation (**X**). The following values were used: P_S(G→H)_ f = 0.95, P_S(G→A)_ f = 0.53, P_S(G→T)_ f = 0.276, P_S(G→C)_ f = 0.14, P_S(A→B)_ f = 0.05, P_S(A→C)_ f = 0.01, P_S(A→G)_ f = 0.03, P_S(A→T)_ f = 0 0.01), b = 15241995.5 using a 50% GC content in *A. nidulans* (Galagan *et al.* 2005), m = 105, s is variable and k = 0.5 (50% kill) or 0.9 (90% kill).

The probability of nucleotide saturation of both guanine and adenine is therefore:

$$P_{S\left(G\to H\;\mathrm{and}\;A\to B\right)}=P_{S\left(G\to H\right)}\times P_{S\left(A\to B\right)}$$

And the probability of substitution saturation of both guanine and adenine is as follows:

$$P_{S\left(G\to A,T,C\;\mathrm{and}\;A\to C,G,T\right)}=P_{S\left(G\to A\right)}\times P_{S\left(G\to T\right)}\times P_{S\left(G\to C\right)}\times P_{S\left(A\to C\right)}\times P_{S\left(A\to G\right)}\times P_{S\left(A\to T\right)}$$

## Results and Discussion

### 4-NQO mutations are distributed across the genome

**Figure 1:**
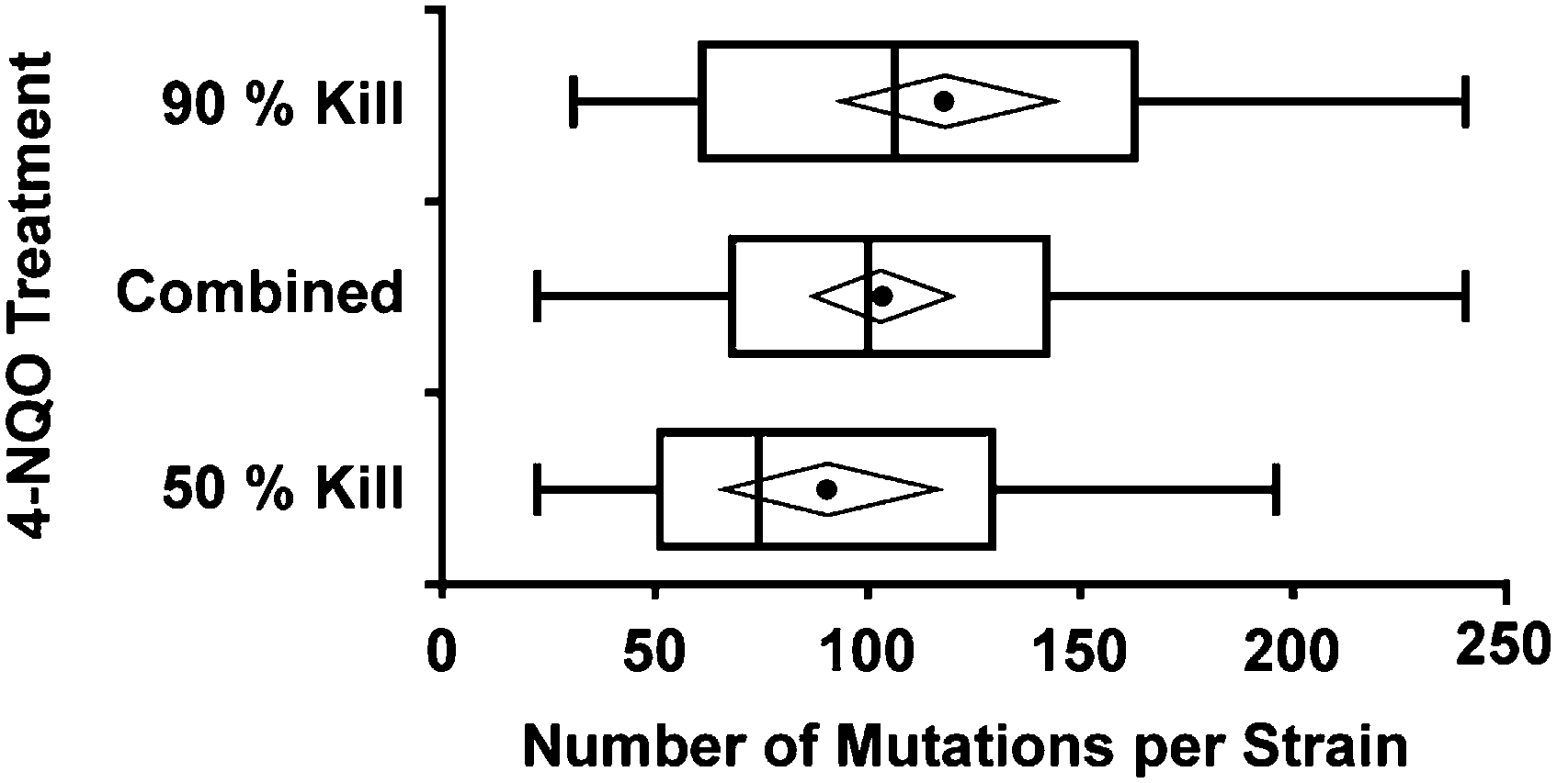
Number of point mutations per strain is not dose-dependent. Distribution of the number of mutations per strain resulting from 50% kill (N = 17; 0.24 µg mL^−1^ 4-NQO per 10^7^ spores) and 90% kill (N = 20; 0.45 µg mL^−1^ 4-NQO per 10^7^ spores) as well as combined data (N = 38). There was no significant difference between the number of mutations induced by 50% kill compared with 90% kill using unpaired unequal distribution Student’s *t*-test. The combined data includes the single mutant from 97% kill (4.0 µg mL^−1^ 4-NQO per 10^8^ spores) with 70 mutations. Boxplots show minimum and maximum (whiskers), median (dividing line), mean (circle), and 95% confidence interval of mean (diamond).

To determine the effects of 4-NQO mutagenesis on *A. nidulans* DNA, we used whole genome sequence data from two independent genetic screens. The first mutagenesis involved direct selection for reversion of a proline nonutilization phenotype conferred by fusion of a nuclear export signal to the transcription factor PrnA (D. J. Downes and R. B. Todd, unpublished data). Mutant strains were generated with a dose of 4-NQO resulting in 97% kill. We isolated nine mutant strains from this screen by direct selection for proline utilization. For eight mutant strains, the causative mutations mapped to the *prnA* locus, whereas for the ninth mutant strain the proline utilization phenotype was unlinked to *prnA*. Mutations in *prnA* were identified by sequencing polymerase chain reaction products (Table 1). The strain containing the unlinked mutation and the mutagenesis parent were used for whole genome sequencing. The second mutagenesis was for a microscopy-based screen for defective organelle transport on rich media (Tan *et al.* 2014). Conidia were treated with doses of 4-NQO conferring 50% or 90% kill. Mutant strains of interest were identified by visual screening for mislocalization of fluorescently labeled nuclei, endosomes and peroxisomes (Tan *et al.* 2014). To identify all lesions induced in this screen bulked segregant progeny of 40 mutant strains, 17 from 50% kill, and 23 from 90% kill, the mutagenesis parent and the outcross parents were sequenced. Reads from both screens were mapped to the *A. nidulans* FGSC_A4 reference genome (Galagan *et al.* 2005), providing sufficient coverage high quality variant calling in all regions excluding centromeres and the nucleolar organizing region ribosomal DNA repeats on Chromosome V (Brody *et al.* 1991; Clutterbuck and Farman 2008). Although our mutant strains were selected or chosen for specific phenotypes and therefore bias may occur for the causative mutation, most of the mutations arising throughout the genome will be random mutations unrelated to the observed phenotypes. Therefore, these mutations represent a data set of 4-NQO−derived sequence changes that are neither biased by selection nor constrained by function. In total we identified almost 7000 mutations in the 41 mutant strains that were absent in the parents. However, ~42% of these mutations were in just three strains. These three mutant strains each carried a substitution or nonsense mutation in at least one DNA repair gene (Supporting Information, File S1). These genes either lacked mutations in the 38 mutant strains with a lower mutation load, or in three cases carried only silent mutations or conservative substitutions. As the mutations arising in the three high mutation load strains may be due to defective DNA repair, rather than resulting directly from 4-NQO−induced mutagenesis, they were excluded from further analysis. Of the remaining 3994 4-NQO−induced mutations distributed across the genomes of 38 mutant strains, 3993 were single-nucleotide substitutions and one was a ∆G:C single base-pair deletion (File S2). The total number of mutations per strain ranged from 23 to 240; however, there was no significant difference in the mutation load arising from different 4-NQO doses and kill percentages (Figure 1). Therefore, we pooled the data for mutants isolated following different mutagen doses for subsequent analyses. The lack of a dose effect on the number of observed mutations per strain in our dataset seems somewhat counterintuitive. It is possible that this could result from the sample size of our data, or our inability to determine the number of mutations in the unrecovered strains killed or selected against.

**Table 1:** 4-NQO mutations selected by phenotype at specific loci in *A. nidulans*

Target(s)	Reference		Number and type of mutation							
		% Kill*^a^*	G → A	G → C	G → T	A → C	A → G	A → T	+N*^b^*	∆N*^c^*
*areA*	Al Taho *et al.* 1984; Kudla *et al.* 1990	99.9			1					
*areB*	Conlon *et al.* 2001	−	1		1					
*cnxE*	Heck *et al.* 2002	−	5		3			1		1
*hypA*	Harris *et al.* 1994; Kaminskyj and Hamer 1998; Shi *et al.* 2004	70	2							
*hypB*	Kaminskyj and Hamer 1998; Yang *et al.* 2008	−		1						
*kinA*, *nudA,F,K*	Tan *et al.* 2014	50, 90	1	1	5					
*meaA*	Monahan *et al.* 2002	−	9	1	2		1		1	2
*nimA*	Wu *et al.* 1998	80−95		1						
*nimA*, *sonA-C*	Larson *et al.* 2014	—	21	9	4					
*nrtA*	Kinghorn *et al.* 2005	—	7	5	5					
*prnA*	Pokorska *et al.* 2000	—	28							
*prnA-areA*^NES^*-gfp*	This study	97			6					2
*sepH*	Harris *et al.* 1994; Bruno *et al.* 2001	70		1						
*swoA*	Harris *et al.* 1994; Momany *et al.* 1999; Shaw and Momany 2002	70			1					
*swoC*	Harris *et al.* 1994; Momany *et al.* 1999; Lin and Momany 2003	70			1					
*swoF*	Harris *et al.* 1994; Momany *et al.* 1999; Shaw *et al.* 2002	70			1					
*swoH*	Harris *et al.* 1994; Momany *et al.* 1999; Lin *et al.* 2003	70			1					
*uaY*	Oestreicher and Scazzocchio 2009	>99	17	8	12			2	1	1
*uaY*	Cecchetto *et al.* 2012	90		16	1	2		3		
Total			91	43	44	2	1	6	2	6

4-NQO, 4-nitroquinoline 1-oxide.

a −, not reported.

b +N, insertion.

c ∆N, deletion.

**Figure 2:**
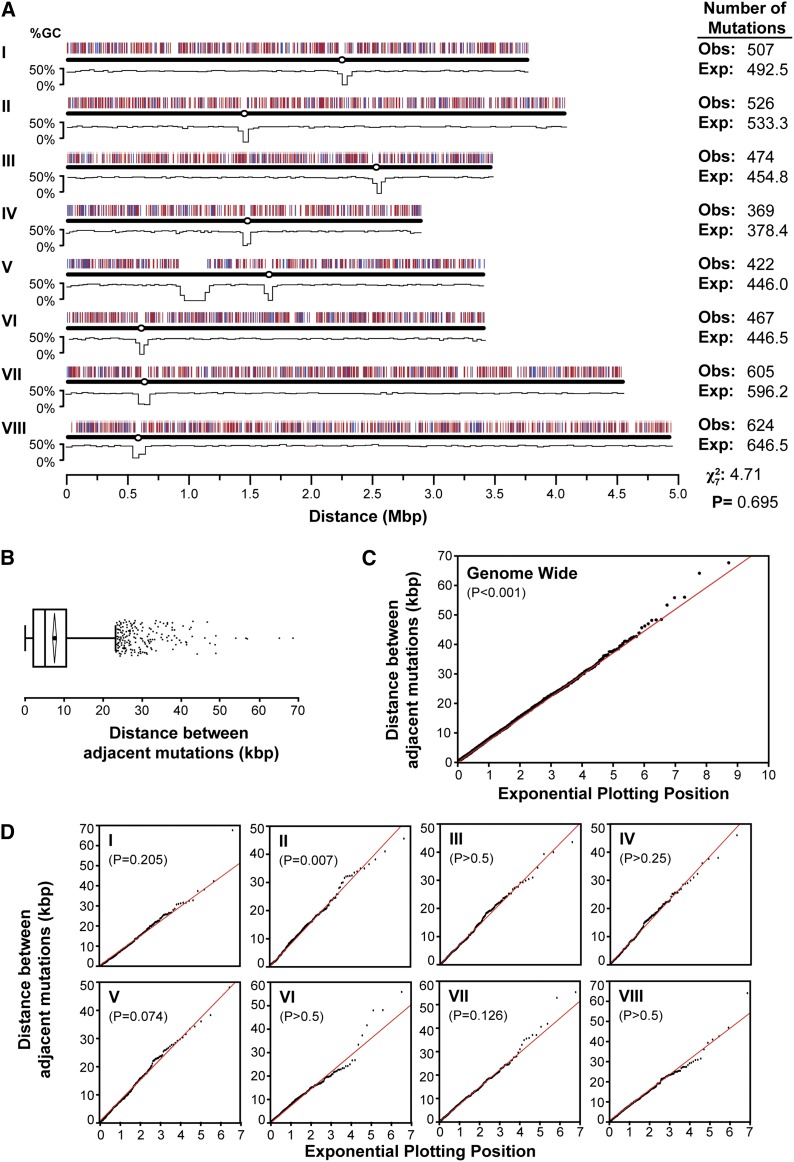
4-NQO mutations are randomly distributed across the genome. (A) *A. nidulans* chromosome map showing locations of 3994 mutations arising from 4-NQO mutagenesis and %GC content (Galagan *et al.* 2005). Mutations within genes (transcribed regions) are red and those outside genes are blue. Centromeres are marked as circles. Expected (Exp.) number of mutations per chromosome was calculated by dividing 3994 by the proportion of genome content in each chromosome. Obs., observed. (B) Boxplot of distance between mutations showing minimum and maximum values within 1.5 × interquartile range of the box (whiskers), median (dividing line), mean (circle), 95% confidence interval of mean (diamond), and outliers (squares). (C−D) Exponential quantile-quantile plot of distances between mutations compared with theoretical exponential distribution (red line) where λ−^1^ = mean. *P*-value shown for Kolmogorov-Smirnov test D statistic. N is the number of distances between mutations. Distances between mutations flanking centromeres and the ribosomal repeats were excluded. Genome (N = 3977, mean = 7461.44, D = 0.0247), I (N = 505, mean = 7332.67, D = 0.0388), II (N = 524, mean = 7610.55, D = 0.0584), III (N = 472, mean = 7226.80, D = 0.0297), IV (N = 367, mean = 7717.14, D = 0.0375), V (N = 419, mean = 7411.85, D = 0.0529), VI (N = 465, mean = 7167.69, D = 0.0251), VII (N = 603, mean = 7397.4, D = 0.0389), VIII (N = 622, mean = 7782.67, D = 0.0201).

To determine whether the effects of 4-NQO are biased toward particular regions of the genome or occur randomly, we classified each of the 3994 mutations as affecting either predicted transcribed regions (5′ untranslated region, coding, intron and 3′ untranslated region sequences) or intergenic regions (all other sequences). We found 2724 mutations within predicted transcribed regions and 1270 mutations in intergenic regions, consistent with relative genome content for each class. The mutations mapped to all regions of the genome, excluding mitochondrial DNA, the centromeres, and ribosomal repeats, where low coverage limited single-nucleotide polymorphism (SNP) calling (Figure 2A). The observed number of mutations per chromosome was not significantly different from that expected, calculated based on DNA content under random distribution (χ^2^ = 4.7, *d.f*. = 7, *P* = 0.695) (Figure 2A). The distances between randomly occurring mutations are expected to follow an exponential distribution with a rate of λ, where λ^-1^ is the mean distance between mutations (Sun *et al.* 2006; Farrell *et al.* 2014). The majority of the mutations were 3−11 kbp apart with a mean spacing of 7461 bp (Figure 2B). An exponential quantile-quantile plot comparing the observed distances between mutations in the whole genome against the expected exponential distribution shows a close match with the theoretical distribution (Figure 2C). However, a one-sample Kolmogorov-Smirnov goodness-of-fit test has a *P*-value < 0.01 (N = 3977, mean = 7,461.44, D = 0.0247) suggesting the observed data differ significantly from the expected trend. To determine whether this was consistent across the genome, we constructed quantile-quantile plots for each of the eight chromosomes (Figure 2D). Like the whole genome data, the observed distribution for each chromosome follows the exponential line closely. For all chromosomes except Chromosome II, the Kolmogorov-Smirnov test statistically supports an exponential distribution. Therefore, the majority of 4-NQO−generated mutations conform to the expected exponential distribution and are randomly distributed. We observed 71 mutations in very close proximity (<10 bp) to another mutation in the same mutant (File S3). These mutations may have arisen either independently from multiple bulky adducts or from a single adduct and an additional repair-based error. Because these two events cannot be distinguished and these mutations comprise <2% of the total data pool, they are considered individual events for all further analyses.

### 4-NQO confers all six possible transitions and transversions

**Figure 3:**
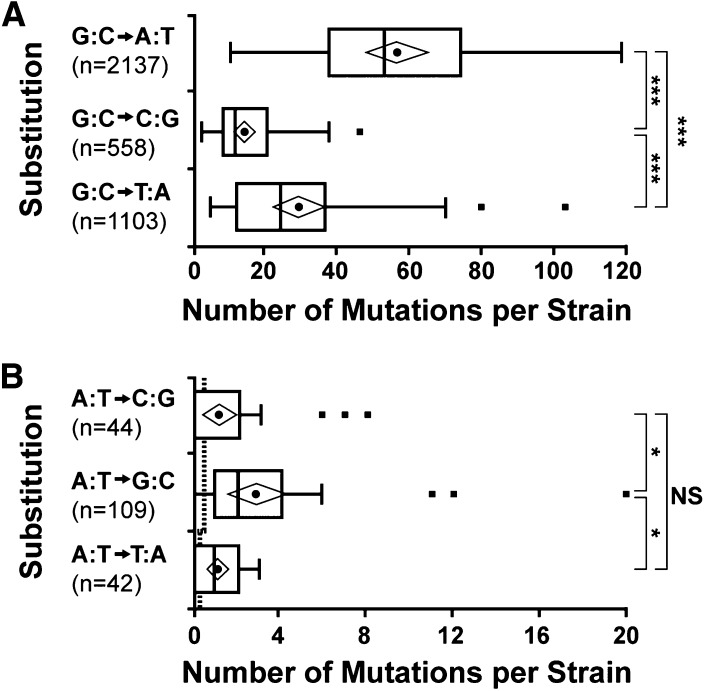
4-NQO induces all six possible base pair substitutions. Distribution of the number of substitutions affecting guanine-cytosine (A) and adenine-thymine (B) base pairs per mutant. Note the different scales on the x-axis for A and B. The dashed line in B shows the predicted number of spontaneous mutations per individual. Boxplots show minimum and maximum values within 1.5 × interquartile range of the box (whiskers), median (dividing line), mean (circle), 95% confidence interval of mean (diamond), and outliers (squares). Using unpaired unequal distribution Student’s *t*-test: NS, not significantly different, *P < 0.05 and ***P < 0.001.

4-NQO was previously reported to induce transitions or transversions of guanine residues and frameshifts in bacteria and yeasts (Prakash *et al.* 1974; Janner *et al.* 1979; Rosenkranz and Poirier 1979). However, adducts of adenine are also formed and therefore adenine is a possible target (Galiègue-Zouitina *et al.* 1984, 1985; Bailleul *et al.* 1989; Menichini *et al.* 1989). Of the 3994 mutations identified from our screens, 3799 (95.12%) resulted from mutation of a guanine and only 195 (4.88%) from mutation of an adenine, consistent with the preference for guanine adduct formation (Figure 3, A and B). For SNPs of both guanine and adenine transition mutations were more frequent than transversions, with 56.27% (2137/3798) transitions for guanine (χ^2^ = 59.65, *d.f*. = 1, *P* < 0.0001) and 55.90% (109/195) transitions for adenine (χ^2^ = 2.71, *d.f*. = 1, *P* = 0.099). The most common mutation was G:C to A:T. Conversion of G:C to T:A, or conversion of G:C to C:G occurred at intermediate frequencies (Figure 3A). Mutation of A:T was rare (<5%) and in some individual mutant strains was not detected, but all three possible substitutions were observed in the complete data set (Figure 3B). To ensure the low frequency of adenine mutations was consistent with chemical mutagenesis rather than spontaneous mutation, we estimated the predicted level of spontaneous changes. Although studies of spontaneous mutation rate have been carried out in *A. nidulans*, they provide rates only for specific loci and not the whole genome (Lilly 1965; Alderson and Hartley 1969; Babudri and Morpurgo 1990; Baracho and Baracho 2003). Spontaneous mutation rates are very similar in *Aspergillus* spp., *Neurospora crassa*, and *S. cerevisiae* (Drake *et al.* 1998). Using an estimate of 0.0034 mutations per replication (Drake *et al.* 1998) with 30 days active growth between mutagenesis and sequencing and 1 hr per nuclear division (Bainbridge 1976), we predict an average of 2.5 spontaneous mutations may have arisen per strain. Similarly, calculations using sequence length and number of generations based on two whole genome studies in *S. cerevisiae* (Lynch *et al.* 2008; Zhu *et al.* 2014) predict just 3.5 spontaneous mutations per strain. By distributing the number of predicted spontaneous mutations across the six possible changes at the ratio described in the whole genome studies (Lynch *et al.* 2008; Zhu *et al.* 2014), we found all three types of A:T substitutions were more frequent than the expected spontaneous mutation level (Figure 3B). Therefore 4-NQO mutagenesis can cause all possible single-nucleotide substitutions. In previous 4-NQO mutagenesis studies using tester strains, mutations of adenine were reported as either absent (Prakash *et al.* 1974) or low-frequency events (~7%) and were only significantly different to nonmutagenized control strains in three of six experiments (Janner *et al.* 1979). We found only one occurrence of a deletion and no insertions. This low indel frequency suggests that this mutation may have arisen spontaneously. Therefore, we found no evidence for 4-NQO−induced frameshift mutations.

### 4-NQO−induced mutations are not influenced by nucleotide flanking sequence

For some mutagens, such as UV light and methyl-nitroso urea, the sequence context can influence the outcome of mutagenesis (Kurowska *et al.* 2012; Setlow *et al.* 1963). We analyzed the adjacent sequence for each of the six mutation types using the 10 upstream and 10 downstream nucleotides of all 3993 SNPs (Figure S1). For all six substitutions, there was no consensus outside of the affected residue, suggesting that only the adenine or guanine is required for efficient adduct formation. Therefore, 4-NQO can potentially target any nucleotide pair within the *A. nidulans* genome.

### Phenotype-associated 4-NQO mutation spectrum frequencies differ from nonbiased whole genome data

**Figure 4:**
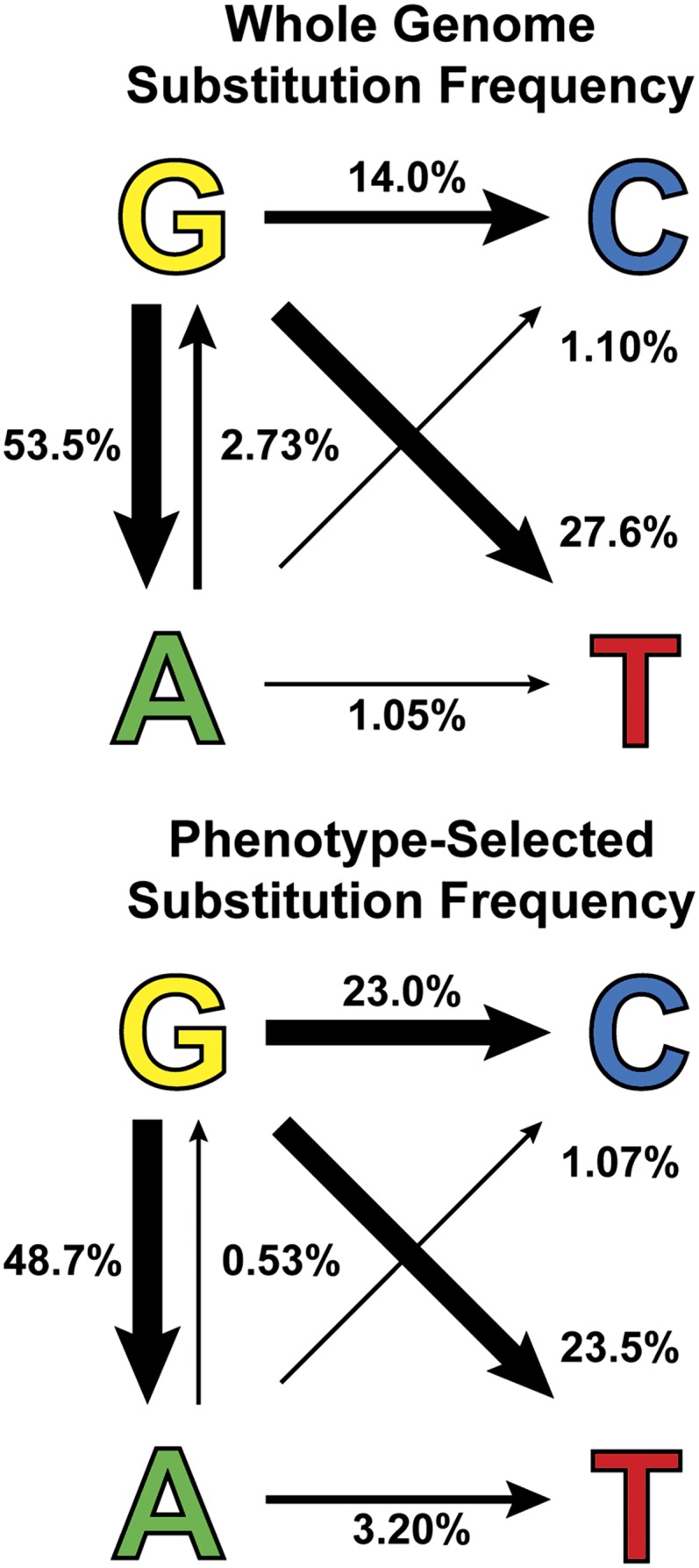
4-NQO affects primarily guanine nucleotides. Relative frequency (percent) of nucleotide substitutions identified by whole genome sequencing of random mutations and in phenotype-selected changes from published screens and this study (Table 1). Weighted arrows indicate change from wild type to mutant nucleotide.

Although mutant strains arising from the screens in this work were selected for specific restoration of proline utilization or defective organelle transport phenotypes, we expect only one or a few of the mutations identified by whole genome sequencing of each mutant strain to contribute to the selected phenotype as causative mutations (Nowrousian *et al.* 2012; Tan *et al.* 2014). Although mutations at some loci will be constrained by function due to their requirement for growth or viability under the selection conditions, normal morphology, or ability to cross for genetic analysis, for example, the majority of mutations are expected to be unrelated to the selection. 4-NQO has been used in many mutagenic screens since being reported as a good mutagen for producing both loss-of-function and altered function mutants in *A. nidulans* (Bal *et al.* 1977). We collated data from the literature and from this study for genetic screens in which mutants were selected for a diverse range of phenotypes and where sequence data were reported or the exact mutation associated with the selected phenotype could be inferred (Table 1). To compare our whole genome mutation frequencies with phenotype-selected mutation frequencies, we used a one-way frequency table with χ^2^ analysis. The distribution of mutation types for the two data sets was significantly different (χ^2^ = 22.50, *d.f*. = 5, *P* = 0.0004). Interestingly, G:C to C:G and A:T to T:A transversions were significantly more common, whereas G:C to A:T and A:T to G:C transitions were less common in the phenotype-selected data compared with the whole genome data set (Figure 4). These differences may be accounted for by the functional constraints of the selection of these mutations. For 24 amino acid codons (those encoding Phe, Leu Tyr, His, Gln, Asn, Lys, Asp, Glu, Cys, Ser, Arg) a transition in the third base position results in a synonymous change unlikely to alter the phenotype, whereas a transversion causes a nonsynonymous change. To test this hypothesis, we performed one-way frequency analysis on the number of transitions and transversions in the two data sets (χ^2^ = 3.60, *d.f*. = 1, *P* = 0.057). Although not significantly different by the conventional 95% confidence level, this test raises the possibility that functional constraints in the selection of mutants could be an important parameter. Therefore, the rates and types of mutations identified by whole genome sequencing of mutants likely approximate the true mutagenic spectrum for survivors of 4-NQO mutagenesis in *A. nidulans*, whereas the historical data are impacted by the constraints of phenotypic selection at the specific loci studied.

### Prediction of 4-NQO screen saturation

**Figure 5:**
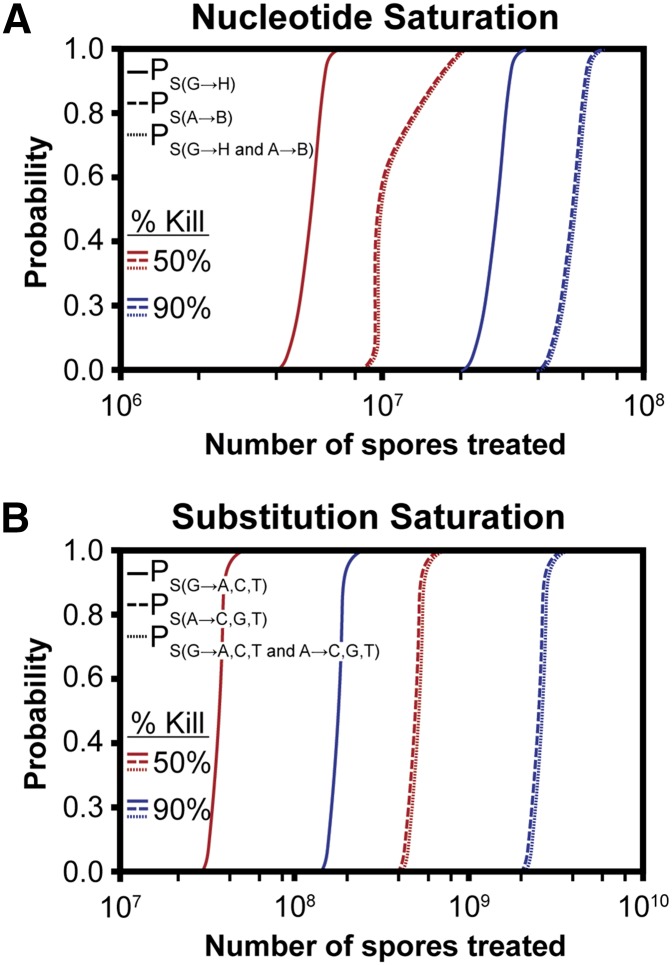
Number of spores required for screen saturation. (A) Probability of mutating every G:C (solid), A:T (dashed), and every nucleotide (dotted) in the *A. nidulans* genome at least once (nucleotide saturation) with 4-NQO doses causing 50% (red), and 90% kill (blue) were calculated using a random sampling with replacement equation. Note, P_S(G→H and A→B)_ = P_S(A→B)_ as for this number of treated spores P_S(G→H)_ = 1. (B) The same equation was used to calculate the number of spores required to generate every possible substitution at every nucleotide (substitution saturation) under the same conditions. Note, P_S(G→A,C,T and A→C,G,T)_ = P_S(A→C,G,T)_ as for this number of treated spores P_S(G→A,C,T)_ = 1.

The purpose of a genetic screen is to identify genes contributing to a particular phenotype. Generally, a screen that has identified every gene associated with a pathway or phenotype is considered a saturation screen, as was most elegantly demonstrated in the seminal *Drosophila melanogaster* developmental screen carried out by Nüsslein-Volhard and Wieschaus (1980). Even though estimating the number of possible genes involved in the pathway or phenotype is difficult, several methods, which use gamma or Poisson distributions, have been used to predict gene saturation (Pollock and Larkin 2004). Our whole genome characterization of 4-NQO mutagenesis identified both the mean number and relative frequencies of nucleotide substitutions and therefore allows prediction of the probability of saturation by using a random sampling with replacement equation (see the section *Materials and Methods*). Our approach calculates the number of spores required to mutate every nucleotide (nucleotide saturation), which is an overestimate of the number of spores required to reach gene saturation. Using our equation, we calculate 2 × 10^7^ or 1 × 10^8^ spores with a kill of 50% and 90%, respectively, are sufficient to isolate a mutation in every A:T and G:C pair and in effect reach nucleotide saturation (Figure 5A).

How many spores would need to be used to isolate every possible mutation at every possible site? Using the same equation, we determined the number of spores required to generate every possible substitution at every nucleotide (substitution saturation). Interestingly, only 4 × 10^7^ spores are required with a 50% kill to reach substitution saturation for guanine, and only 15 times as many spores (6 × 10^8^) are required to reach substitution saturation of both guanine and adenine (Figure 5B). Using a 90% kill, substitution saturation of guanine can be achieved with 2 × 10^8^ spores; however, 4 × 10^9^ spores are required to saturate adenines. Current 4-NQO mutagenesis protocols in *A. nidulans* use between 10^7^ and 10^8^ spores, and therefore easily reach nucleotide saturation or even substitution saturation. Many laboratories use alternative physical or chemical mutagenesis methods for *A. nidulans*, including UV light and MNNG. It will be interesting to use the approach we used here to do a comparative study of the outcomes and efficacy of these mutagens.

Mutant screens in *A. nidulans* to characterize diverse cellular processes, including metabolism, mitosis, and organelle transport have used the highly carcinogenic chemical mutagen 4-NQO to induce sequence changes. Using a whole genome approach, we have characterized the mutagenic spectrum of 4-NQO and determined that its effects are distributed across the genome in a manner unbiased by sequence other than a preference for guanine over adenine at a ratio of 19:1. Interestingly, 4-NQO dose did not impact the number of mutations caused within a single surviving strain for 50% and 90% kill percentages. Therefore, future screens and kill percentages can be designed to suit whether selection or manual screening is required to identify a trait of interest. The number of mutations ranged between 23 and 240 per mutant. Importantly for *A. nidulans* mutant screens, this is a manageable number of candidate mutations to test for causation of the selected phenotype when combined with the power of haploidization and/or meiotic mapping, or with bulk segregant analysis. Additionally, we have shown that all six possible sequence transitions and transversions are induced by 4-NQO adduct repair, making it possible to conduct saturation screens with this chemical. We conclude that current practices using 4-NQO mutagenesis are sufficient to reach gene saturation in genetic screens. Therefore, our findings provide genome-wide evidence for the assertion of Bal *et al.*, (Bal *et al.* 1977) that “4-NQO is a good mutagen for *A. nidulans*.”

## Supplementary Material

Supporting Information
