# Supplementary material for: Characterization of the Mutagenic Spectrum of 4-Nitroquinoline 1-Oxide (4-NQO) in Aspergillus nidulans by Whole Genome Sequencing
Source: G3 (Bethesda). 2014 Oct 27;4(12):2483–92. doi: 10.1534/g3.114.014712 (PMC4267943; doi:10.1534/g3.114.014712)
Supplement: Supporting Information [file supp_4_12_2483__index.html]

Characterization of the Mutagenic Spectrum of 4-Nitroquinoline 1-Oxide (4-NQO) in Aspergillus nidulans by Whole Genome Sequencing — Supporting Information 

# Characterization of the Mutagenic Spectrum of 4-Nitroquinoline 1-Oxide (4-NQO) in *Aspergillus nidulans* by Whole Genome Sequencing

## Supporting Information for Downes *et al.*, 2014

**Files in this Data Supplement:**

- Figure S1 - 4-NQO acts in a sequence context independent manner. (PDF, 65 KB)
- File S1 - Mutations in DNA repair genes identified in mutants with very high mutation load. (.xlsx, 28 KB)
- File S2 - Characteristics of mutations identified in this study. (.xlsx, 349 KB)
- File S3 - Characteristics of mutations identified in close proximity in the same mutant. (.xlsx, 57 KB)
