# Supplementary material for: Characterization of the Mutagenic Spectrum of 4-Nitroquinoline 1-Oxide (4-NQO) in Aspergillus nidulans by Whole Genome Sequencing
Source: G3 (Bethesda). 2014 Oct 27;4(12):2483–92. doi: 10.1534/g3.114.014712 (PMC4267943; doi:10.1534/g3.114.014712)
Supplement: Supporting Information [file supp_g3.114.014712_FigureS1.pdf]

Downes *et al.*, Characterization of the mutagenic spectrum of 4-nitroquinoline 1-oxide (4-NQO) in *Aspergillus nidulans* by whole genome sequencing.

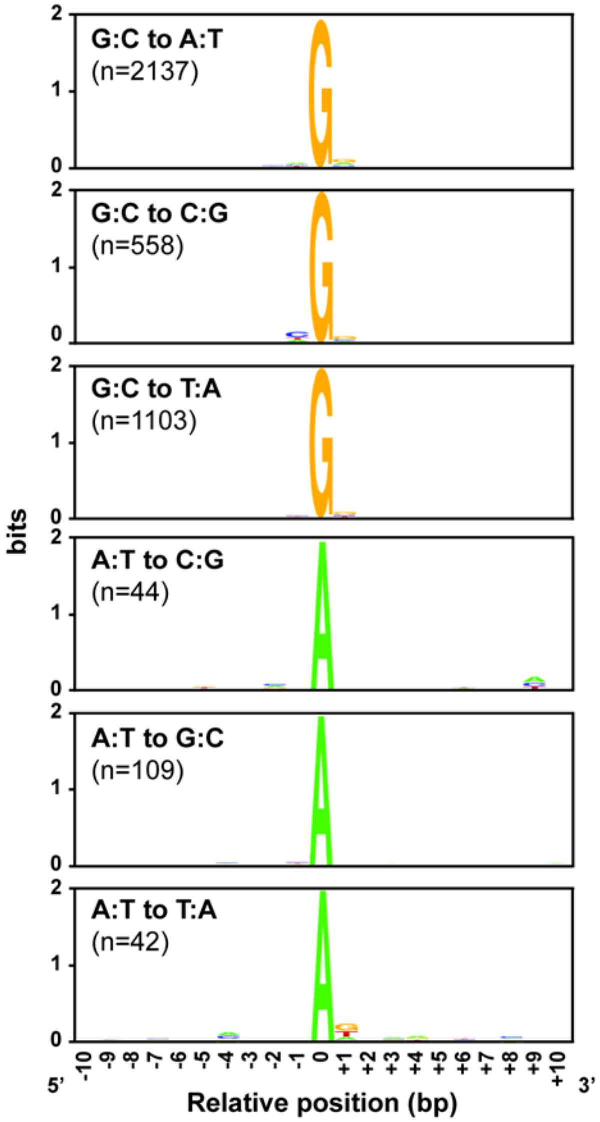

Figure S1 4-NQO acts in a sequence context independent manner.
Consensus sequence for each nucleotide substitution including 10 bp upstream and 10 bp downstream. Units measure bits of information proportional to sequence conservation.
